# Long-Lasting Changes in DNA Methylation Following Short-Term Hypoxic Exposure in Primary Hippocampal Neuronal Cultures

**DOI:** 10.1371/journal.pone.0077859

**Published:** 2013-10-25

**Authors:** Iain Hartley, Fuad F. Elkhoury, Joo Heon Shin, Bin Xie, Xiangqun Gu, Yuan Gao, Dan Zhou, Gabriel G. Haddad

**Affiliations:** 1 Division of Respiratory Medicine, Department of Pediatrics and Biomedical Sciences Graduate Program, University of California San Diego, La Jolla, California, United States of America; 2 Division of Genomics, Epigenomics and Bioinformatics, Lieber Institute for Brain Development, Baltimore, Maryland, United States of America; 3 Department of Neurosciences, University of California San Diego, La Jolla, California, United States of America; 4 Rady Children’s Hospital, San Diego, California, United States of America; Università di Napoli Federico II, Italy

## Abstract

While the effects of hypoxia on gene expression have been investigated in the CNS to some extent, we currently do not know what role epigenetics plays in the transcription of many genes during such hypoxic stress. To start understanding the role of epigenetic changes during hypoxia, we investigated the long-term effect of hypoxia on gene expression and DNA methylation in hippocampal neuronal cells. Primary murine hippocampal neuronal cells were cultured for 7 days. Hypoxic stress of 1% O2, 5% CO2 for 24 hours was applied on Day 3, conditions we found to maximize cellular hypoxic stress response without inducing cell death. Cells were returned to normoxia for 4 days following the period of hypoxic stress. On Day 7, Methyl-Sensitive Cut Counting (MSCC) was used to identify a genome-wide methylation profile of the hippocampal cell lines to assess methylation changes resulting from hypoxia. RNA-Seq was also done on Day 7 to analyze changes in gene transcription. Phenotypic analysis showed that neuronal processes were significantly shorter after 1 day of hypoxia, but there was a catch-up growth of these processes after return to normoxia. Transcriptome profiling using RNA-Seq revealed 369 differentially expressed genes with 225 being upregulated, many of which form networks shown to affect CNS development and function. Importantly, the expression level of 59 genes could be correlated to the changes in DNA methylation in their promoter regions. CpG islands, in particular, had a strong tendency to remain hypomethylated long after hypoxic stress was removed. From this study, we conclude that short-term, sub-lethal hypoxia results in long-lasting changes to genome wide DNA methylation status and that some of these changes can be highly correlated with transcriptional modulation in a number of genes involved in functional pathways that have been previously implicated in neural growth and development.

## Introduction

During early mammalian development, oxygen plays a vital role in the growth and maturation of every organ system. The central nervous system is particularly dependent on oxygen for proper development and function. Hence, a hypoxic insult during development may cause significant cognitive and behavioral impediments at a later stage in life. For example, in the United States, perinatal asphyxia has an incidence of up to 8 per 1000 live births with results ranging from mild neurological difficulties to severe neonatal encephalopathy, including cerebral palsy [1]. Interestingly, these neurological diseases are not always easily correlated to hypoxia-induced neuronal injury [2]. This raises the question of how such temporary hypoxia influences long-term function of cells still viable after hypoxic stress.

While the detrimental effect of short-term hypoxia on neurological development has been phenotypically characterized, the underlying mechanisms responsible for the deleterious effects continue to be elucidated. Furthermore, although it is possible that severe hypoxia in early life can cause cell death, it is also possible that hypoxia can induce sub-lethal biochemical changes, including long-lasting alterations in gene expression. It has been shown that hypoxia activates certain genes necessary for cellular survival and adaptation in hypoxic conditions as well as genes for inducing cell injury [3-5]. For example, acute modest hypoxia, in particular that lasting for minutes or hours, is known to upregulate genes coding for neuroprotective endoplasmic reticulum proteins, proteins involved in ubiquitinylation, and those involved in hypoxic neuronal death[4,6]. Concordantly, genes involved in cellular protection, neurogenesis, and neuronal regeneration were strongly repressed following such a stress [7-9]. Whether these gene expression changes persist many days after the short-term hypoxic stress has yet to be determined.

Epigenetic mechanisms, including DNA methylation and histone modification, result in long-term changes in gene expression [10]. Epigenetic changes are central in controlling longer term effects of stresses such as in hypoxia-mediated gene expression [11,12]. How acute hypoxic stress influences gene expression through modifications of DNA methylation and histone acetylation has been studied in the context of tumor suppressor genes and cancer progression [13]. Other research has demonstrated that intermittent hypoxia may initiate epigenetic changes leading to long-lasting increases in persistent oxidative stress and manifestations of cardiovascular disease in adult rats [14]. However, the enduring effects and the extent of short-term hypoxia on methylation and subsequent gene expression in the central nervous system have not been explored. Thus, in this present study, we investigate the long-lasting impact of acute sub-lethal hypoxic stress on gene expression in hippocampal neuronal cells and hypoxia-induced changes in DNA methylation.

## Materials & Methods

### Ethics Statement

This study was carried out in strict accordance with the recommendations in the Guide for the Care and Use of Laboratory Animals of the National Institutes of Health. The protocol was approved by the Institutional Animal Care and Use Committee of the University of California, San Diego.The mice were anaesthetized with Isoflurane by inhalation and sacrificed. All efforts were made to minimize suffering.

### Primary Hippocampal Cell Culture

Time-pregnant female mice were obtained from Charles River (CD-1 strain). Dissection and culturing protocol was adapted from Fath et. al., 2008 [15]. Briefly, hippocampi were carefully dissected from E16.5 mouse embryo brain. The neuronal cells were dissociated with trypsin (Gibco) digestion. The dissociated cells were re-suspended in neurobasal medium (Gibco) (B27 1× final, Gibco) containing 2mM GlutaMAX. Cells were diluted to 106 cells/ml. 3 milliliters (about 300 cells) of cell suspension were added to each 35-mm poly-d-lysine coated plate and cultured for 2 hours in 5% CO2 at 37°C. The plating medium was then replaced with 3 ml of neurobasal/B27 medium, and the culture was continued in 5% CO2 and 21% O2. The primarily cultured cells were allowed to grow for 48 hours before treatment. 

The morphology of all cultured cells was assessed with microscopy, and cell death was evaluated by trypan blue exclusion assay. Images were acquired with a Zeiss microscope with AxioCam MRm camera using the Axiovision Rel 4.5 software, which was also used to determine the dendrite length and cell area before and after hypoxia treatment.

### Experimental Design

The primary neuronal cells were cultured in room air condition for 48 hours, and then the cultured cells were treated with 1% O2 for 24 hours and returned to room air condition for continuing culture for another 4 days. Cells were harvested at this time point. Genomic DNA was extracted using DNeasy Blood and Tissue Kit (Qiagen, Valencia, CA) to survey CpG methylation using MSCC assay. Total RNA was isolated using RNeasy Mini Kit (Qiagen, Maryland) for RNA-Seq based transcriptome profiling.

The duration and severity of hypoxia was determined prior to the experiment by exposure of different hippocampal cell cultures to varying levels of oxygen during cell incubation. After 48 hours of growing at room air condition with 21% O2 and 5% O2, the cells were transferred to hypoxia chambers and treated with 1%, 2%, or 3% O2 for 24 or 48 hours, respectively. Cell death and morphological changes were assayed immediately after each treatment with trypan blue exclusion assay and microscope imaging. In addition, cellular hypoxic response was monitored by measuring transcriptional changes of hypoxia responding genes (i.e., Vegf and Epo) using quantitative real-time PCR.

Sequenced reads for both RNA-Seq and MSCC assays can be accessed via the NCBI Sequence Read Archive under accession number SRP028757. 

### Genomic wide CpG methylation profiling with Methyl-Sensitive Cut Counting (MSCC)

Genome-wide cysteine methylation profiling was carried out using methyl sensitive cut counting (MSCC) assay as previously described [16,17]. Briefly, the sequencing libraries were generated using 0.65μg of genomic DNA for each library. A methylation sensitive library and an inverse methylation insensitive library were generated for each sample. The methylation sensitive and inverse insensitive libraries were each digested with HpaII and MspI (NEB, Ipswich, MA). An internal CpG methylation standard was created by mixing methylated and unmethylated PCR products of *E. coli* DNA and included in each library generation to represent 0% methylation, 33% methylated, 67% methylated and 100% methylated CpG sites. The CpG tag libraries were sequenced using Illumina Genome Analyzer IIx and returned ~1 GB of sequence partitioned into 36-nt long reads per library. The resulting sequence files were mapped to the *Mus musculus* reference genome (version mm9), and the data were analyzed by creating frequency histograms showing the number of times reads identified at any given CpG site. The CpG sites with at least 30 reads for both methylation sensitive and insensitive libraries combined were included for further analyses to minimize technical variations in each sample, a list of which may be found in Table S1. 

### Transcriptome profiling with RNA-Seq

Each RNA-seq library was generated using 200ng of rRNA-depleted RNA with the Illumina®-compatible ScriptSeq™ mRNA-Seq Library Preparation Kit (Epicentre Biotechnologies, Madison, WI) according to manufacturer’s instruction with 200-250bp insertion fragments. The quality of the libraries was checked on the Bioanalyzer using a High Sensitivity DNA Chip (Agilent, Waldbronn, Germany) and quantified using the Illumina Genome Analyzer DNA library quantification kit (Kapa Biosystems, Woburn, MA). Two libraries for each condition (control and hypoxia-treated) were sequenced using a 2x36 cycle paired-end run on Illumina Genome Analyzer IIx. The resulting reads were mapped using the RUM alignment package [18] with default settings to the mouse reference genome mm9. The aligned reads were then processed with htseq-count to count the number of reads mapped to genes defined by Illumina's iGenome GTF annotation for mm9 (Illumina). The differentially expressed genes were determined by DeSeq [19] wherein variation in the raw read counts was estimated using a technique based on the negative binomial distribution. Results produced by DeSeq were filtered to select genes with a p-value less than 0.05 and an absolute fold change greater than 1.5, a list of which may be found in Table S2.

### Association between changes of CpG methylation in the promoter regions and gene expression

The promoter region of a gene was defined as 1500bp upstream to 500bp downstream of the gene's transcription starting site (TSS). The differentially expressed genes and the changes in CpG methylation that were identified with aforementioned filtering criteria were merged to determine the association between CpG methylation and gene expression. Further filtering was imposed to exclude all the genes with promoter regions containing less than two CpG sites measured to reduce the incidence of noise. The promoter regions of the remaining genes were profiled to determine the direction of methylation change at each site, whether positive, indicating hyper-methylation relative to control, or negative, indicating hypo-methylation relative to control.

## Results

We first present the morphologic changes that occur with hypoxia; we then present the effect of hypoxia on gene expression, and finally the DNA methylation results.

**Figure 1:**
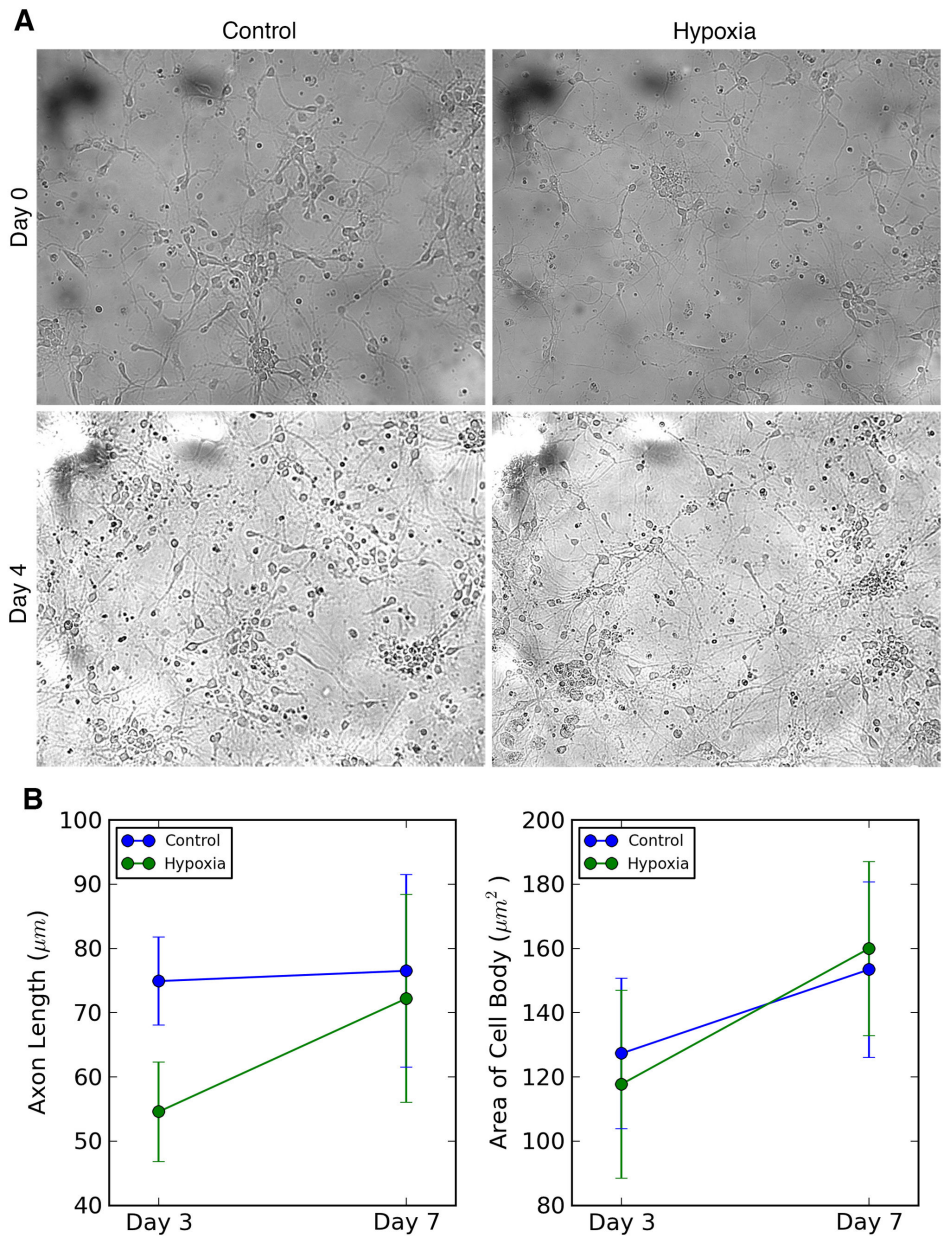
Dendrite growth blunted following 24 hour hypoxic exposure mounts recovery following return to room air. (a) Microscopy of cell cultures immediately following removal of hypoxic sample from the hypoxic incubator in addition to 4 days after return to room air. (b) Quantification of dendritic length and cell area changes both immediately after 24 hours of 1% O2 hypoxic stress (Culture Day 3) and 4 days post return to room air (Culture Day 7).

The hypoxic condition of 1% O2 for 24 hours was found, after a number of trials at various concentrations and durations, to induce a cellular stress response with no or very little cell death (see Methods). It was found that after 24 hours of hypoxic stress, the neuronal dendritic lengths were significantly shorter than the dendritic lengths of the control cells (Hypoxic cells: 54.57 +/-7.78 μm; Normoxic Cells: 74.92 +/-6.85 μm) (Figure 1a,b). However, when the cell cultures initially exposed to hypoxia were later subjected to a normal oxygen environment for 4 days, their dendritic lengths grew rapidly to catch up with the dendritic lengths of the control cells (Hypoxic cells: 72.20 +/- 18.18 μm; Normoxic cells: 76.52 +/- 15.00 μm). Comparing cell body area between the hypoxic and control cells revealed no difference between these two cell populations, both immediately (1 day after exposure to hypoxia) and after 4 days in normoxia (Figure 1b). 

**Figure 2:**
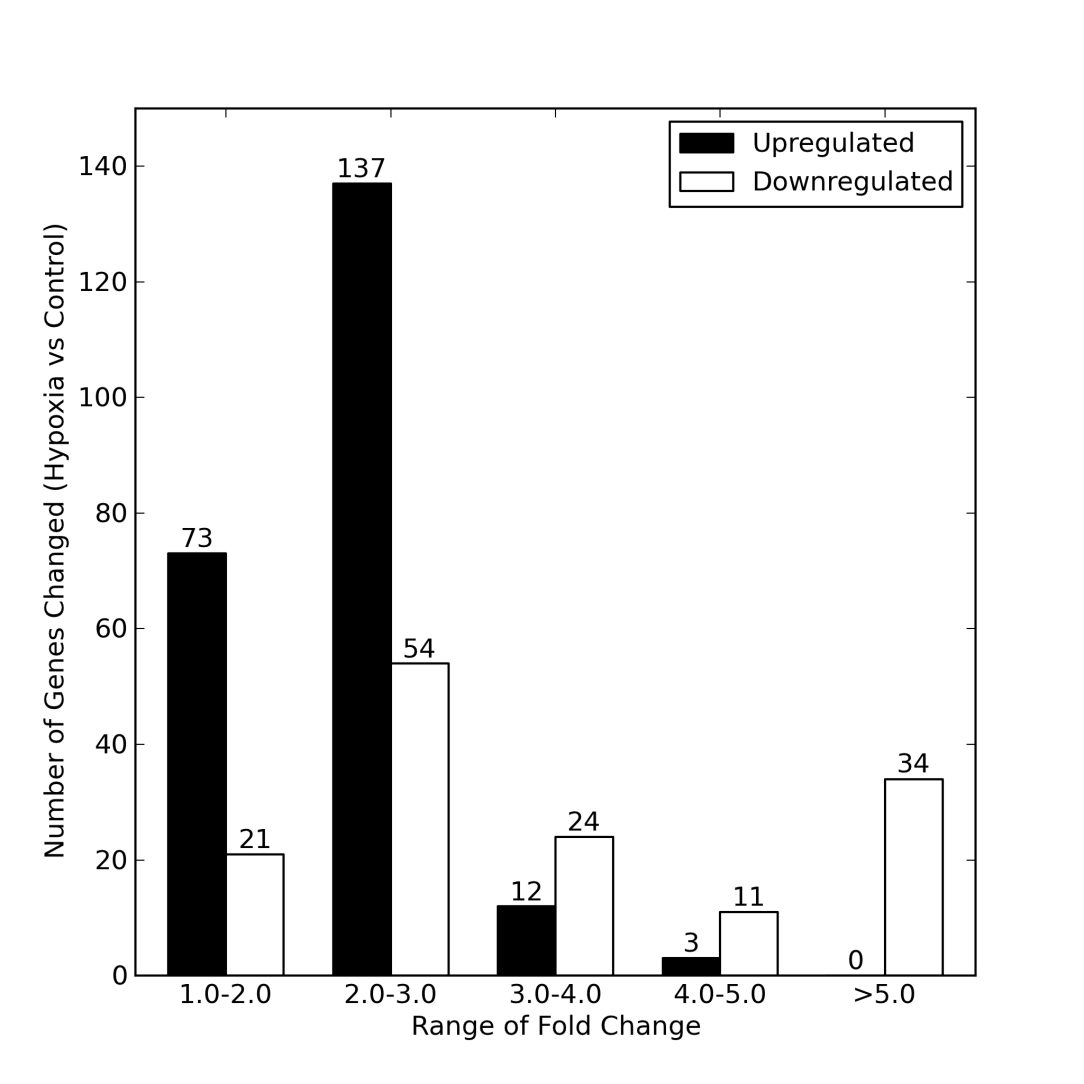
Number of genes with significant modification in expression as determined by RNASeq analysis. RNA isolated from post-hypoxic and control cell cultures was sequenced, the sequence was aligned and the alignment quantified as described in the methods. Each range of fold-change values includes only genes with a p-value less than 0.05.

We next investigated the effects of such short-term hypoxia at the transcriptional level. RNA-Seq revealed that 369 genes were differentially expressed (>1.5-fold change) in the hypoxic cells 4 days after hypoxic exposure was removed compared to control cells. Of these 369 genes, 225 genes were upregulated and 144 genes were downregulated (Figure 2).

**Figure 3:**
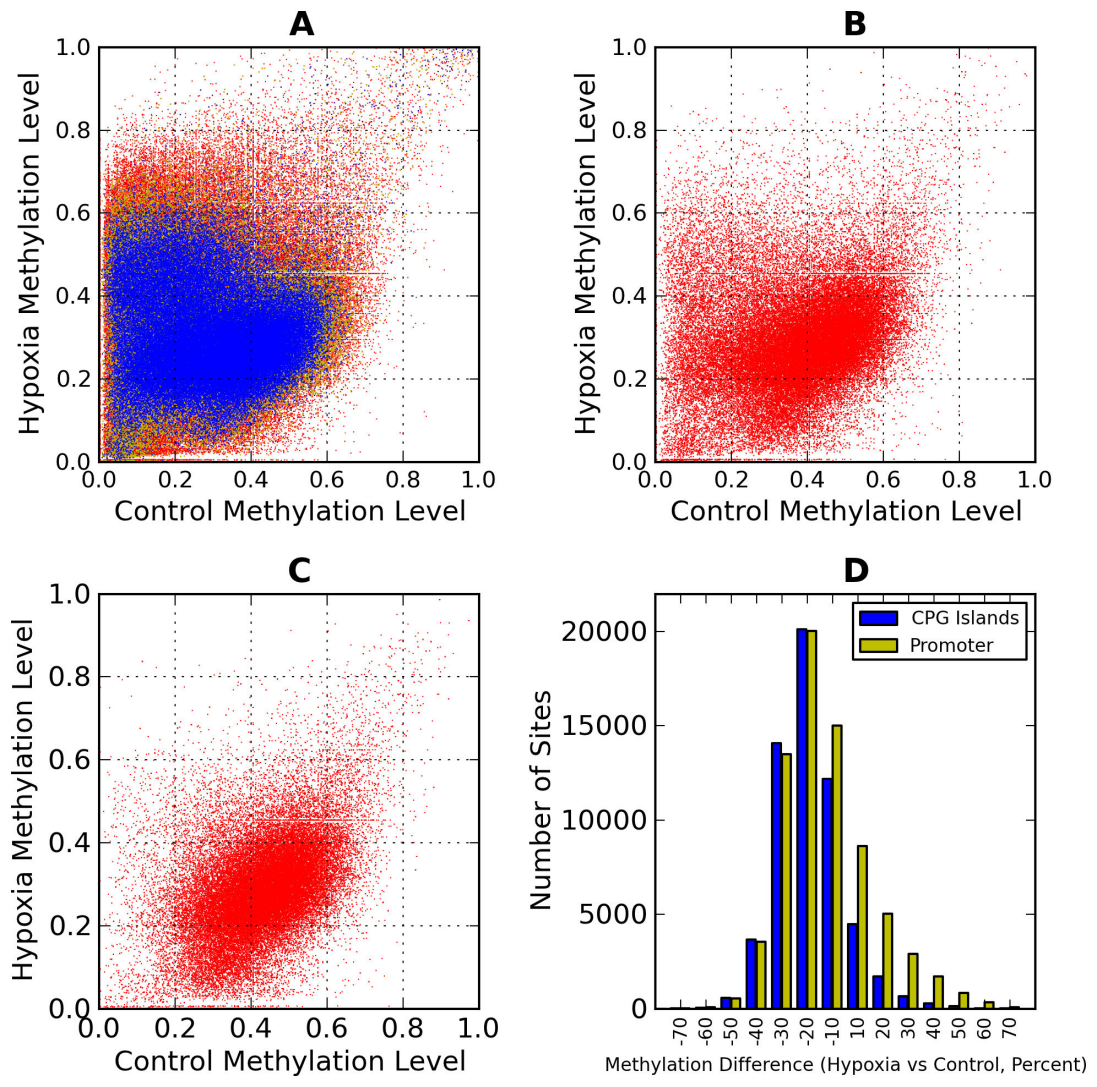
Genome wide methylation assay of the *Mus Musculus* genome. DNA extracted from post-hypoxic and control cell cultures underwent a Methyl Sensitive Cut Counting assay as described in the methods. Vertical axis signifies average methylation of hypoxic sample while the horizontal axis signifies average methylation of control sample in all sub-figures. (a) Each point represents a measured site (CCGG) that conforms to the following conditions: greater than 30 reads in red, greater than 60 reads in green and greater than 100 reads in blue. (b) All measured methylation sites with greater than 30 reads in addition to falling within the promoter region of a gene (Defined as 1500bp upstream of Transcriptional Start Site (TSS) to 500bp downstream of the TSS.) (c) All measured sites that fall within a CpG island in addition to having more than 30 reads at the given locus. (d) Observed sites with various levels of methylation change in Promoter regions and CpG islands.

Analysis of the global methylation profile of all observed CpG sites with greater than 30 reads reveals that out of 268,479 reads (Figure 3a), there is an almost even split between hyper- and hypo-methylated sites with 147,052 exhibiting hypermethylation and the remaining 121,427 displaying hypo-methylation. However, when setting a cutoff to exclude all sites with less than 20% methylation change, 65,653 sites were observed to be hypermethylated and about half of these (30,412 sites) were hypomethylated in hypoxic cells compared to control. Additionally, when changes within gene promoter regions were analyzed, we found that more sites within these regions were hypomethylated following hypoxia with 17,675 of 23,552 observed in gene promoters displaying hypomethylation (Figure 3b). The level of methylation within CpG islands throughout the genome also showed a greater level of hypomethylation in the hypoxic cells compared to control with 50,686 out of 57,976 observed sites displaying hypomethylation (Figure 3c). Importantly, when comparing the data within Figure 3(a-c) it is evident that the majority of hypomethylated sites are localized in gene promoter regions and CpG islands, both regions shown to have significant regulatory impact [20-24]. In contrast, while there are more hypermethylated sites observed to have substantial changes, there appears to be no localization to well-known regulatory regions (Figure 3d).

**Figure 4:**
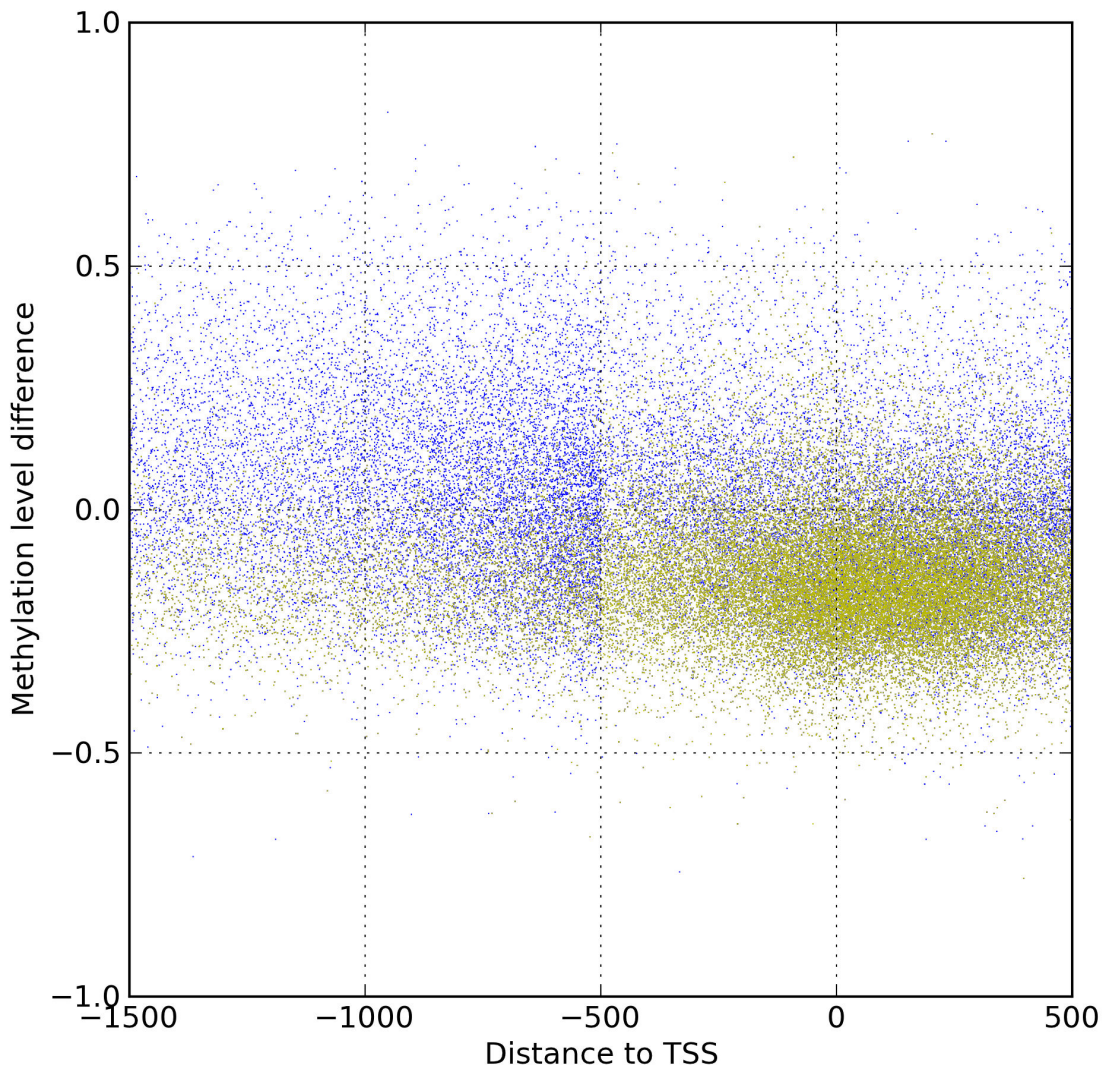
Methylation change relative to nearest Transcriptional Start Site (TSS). Each point represents a methylation site as measured by MSCC analysis and that passed the filters described in the methods section. Vertical axis represents change of methylation in hypoxic treated sample relative to control sample. Horizontal axis is distance from nearest TSS, where negative is upstream and positive is downstream. Green sites fall within a CpG island, in addition to being in the promoter region of a gene.

When we determined the proximity of the methylated CpG sites to the transcription start sites (TSS) of the differentially expressed genes, it was clear that long-term methylation changes due to hypoxia occur predominantly near the transcription start site (Figure 4). Additionally, of the CpG islands undergoing methylation change within 1500bp of a gene TSS, 44,625 of 50,842 observed sites were hypomethylated following acute hypoxic stress. 

**Figure 5:**
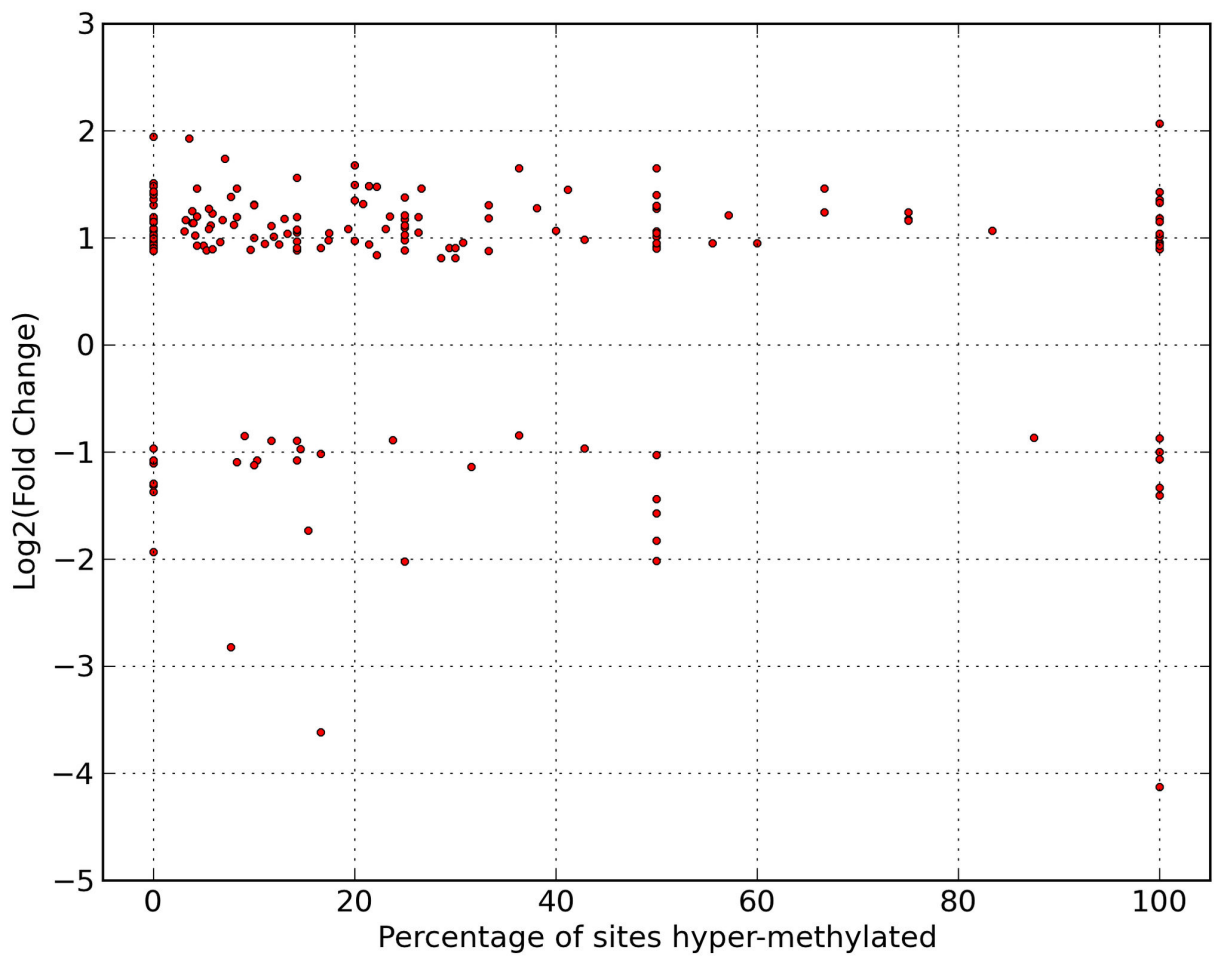
Fold change of differentially expressed genes relative to promoter methylation profile. Each point represents one gene with significant change in expression (p < 0.05) in addition to having at least two measured methylation sites within its promoter region. Vertical axis is log_2_(fold change), horizontal axis is the number of sites displaying hyper-methylation, in the hypoxic sample relative to control, within the promoter region divided by the total number of sites measured expressed as a percentage. Genes exhibiting increased expression with decreased promoter methylation or decreased expression coupled with increased promoter methylation conform to previously described methylation mediated expression regulation while all other genes presumably are regulated by additional epigenetic factors.

We next determined whether the changes in DNA methylation correlated with the changes in gene expression, as expected. It is known that a decrease in DNA methylation in the promoter region is correlated with an up-regulation of the related gene, while an increase in DNA methylation induces the opposite [25,26]. Analysis of the methylation and gene expression changes revealed that 59 genes and their respective changes in DNA methylation followed the aforementioned expected paradigm. More specifically, 53 genes were up-regulated and had hypomethylated promoter regions, and 6 genes were downregulated with hypermethylated promoter regions (using >90% hypermethylated or hypomethylated promoters compared to control as the threshold) (Figure 5). However, there were an additional 22 genes that exhibited the opposite of what was expected, with 12 genes showing up-regulation coupled with hyper-methylated promoters and 10 displaying the down-regulation and hypo-methylated promoters. Pathway analysis of the 59 genes revealed that the top two functional groups characterized contribute to neuronal growth, neurological development, and developmental disorders (p<0.01). Additionally, 4 of the 59 genes with expected correlation between transcript regulation and promoter methylation were found to be involved in the Wnt signaling pathway with the use of the DAVID functional analysis tool (p=1.8e-2) and 3 of the 59 were found to be involved in the mTOR signaling pathway (p=1.8e-2), both of which are implicated in regulation of cell growth. 

## Discussion

We have made several major observations in this study. First, short-term acute sublethal hypoxia caused inhibition of process and dendritic growth of hippocampal neurons, and after return to normal oxygen conditions, cells exhibited a rapid recovery of this growth. Second, this acute hypoxic exposure caused long-term differential expression of 369 genes, of which 225 genes were up-regulated. Third, 59 of the 369 differentially expressed genes displayed modified promoter methylation consistent with the previously observed relation, namely, that decreased promoter methylation correlated with increased RNA transcription and increased promoter methylation correlated to a decrease in transcription. Finally, a whole genomic view of methylation revealed a strong tendency for CpG islands to remain hypomethylated long term after the hypoxic stress was removed. Indeed, short-term, sub-lethal hypoxia results in long-lasting gene expression changes in addition to long lasting and substantial changes to DNA methylation.

Analysis of hippocampal cells exposed to hypoxia revealed blunted growth during stress but accelerated growth following return to normoxia. Previously, other studies have shown that hypoxia indeed suppresses genes involved in neuronal maturation and growth [27,28] but the subsequent phase of catch-up growth observed in this study has never been described in the literature. Short-term hypoxia has been shown to activate genes responsible for hypoxia-induced protection against brain ischemia, though the mechanism through which such hypoxia results in long-lasting tolerance to future hypoxic stress has yet to be elucidated [29-32]. Our results show that DNA methylation plays a lasting role in hypoxia-induced gene expression changes, though the exact nature of this regulatory action is still uncertain. 

In this study, hypoxia resulted in more upregulated (225) than downregulated (144) genes. Also, a greater proportion of promoter and CpG islands were hypomethylated rather than hypermethylated, several days after hypoxic exposure. These findings indicate that there is a correlation between whole genome changes in methylation and gene expression, days after sub-lethal hypoxic stress. When investigating the site of methylation in the 59 genes, it was evident that methylation as an epigenetic mechanism was strongly responsible for the observed lasting changes in gene expression. While these 59 genes fit the widely-accepted paradigm of the influence of methylation on gene expression, the majority of genes that had altered expression either did not have observed methylation changes within their promoter or displayed methylation modification that were contrary to the expected paradigm. This suggests that, while methylation plays a potential role in gene regulation, it is not the only mediator, and there is still much to be discerned regarding the role of methylation in gene expression regulation. 

Transcription regulatory mechanisms are diverse and influenced by various environmental and chemical conditions. Histone acetyltransferases and deacetylases have been found to have a role in HIF1-dependent transcriptional induction [33-37]. Hypoxia-related microRNAs have also surfaced as important in processes such as apoptosis and proliferation [38,39]. Ostensibly, epigenetic regulation of gene expression is rarely a uni-factorial mechanism influencing transcription. While one epigenetic mechanism may play a dominant role in regulating gene expression, there are many other epigenetic influences that may impact transcription. We found that variable expression of 59 genes was predominantly impacted by DNA methylation changes due to sublethal hypoxia. However, there were many other genes whose expression changes are not solely explained by methylation changes. Other epigenetic mechanisms are likely involved in these cases.

Of the 59 genes predominantly impacted by DNA methylation, 4 of them that displayed upregulation of RNA and demethylated promoter regions (APC, CTNNBIP1, CCND2, PLCB1) were found to be involved in the Wnt signaling pathway. APC and CTNNBIP1 act as negative regulators of the canonical pathway through disruption of β-catenin nuclear localization [40,41]. PLCB1 interacts with the Ca2+ pathway and is involved in the development of normal cortical circuitry; mice without PLCB1 display changes in synaptic and dendritic morphology [42]. CCND2 encodes the Cyclin D2 protein involved in cell cycle regulation. Furthermore, Wnt is involved in the up-regulation of neuronal stem cell growth through HIF-1a signaling [43]. 

 We found that RICTOR, a subunit of the mTORC2 complex in the mTOR pathway, was found to have undergone both transcriptional up-regulation and substantial promoter de-methylation in the culture subjected to hypoxia (Table 1). However, Igf1, which has been characterized as an upstream regulator of the mTOR pathway, was found to be down-regulated with increased promoter methylation. While the functional characteristics of mTORC2 have yet to be fully elucidated, loss of mTORC2 has been implicated in impairment of long term memory and long term potentiation and plays a role in cytoskeleton organization [44]. We hypothesize here that this up-regulation of mTORC2 is associated with the increased growth of dendrites with the return to room air after the hypoxic stress.

**Table 1:** Genes with significant changes in expression in addition to having significant methylation changes in their associated promoter regions.

Chr	Name	Fold Change	Sites	% Methylated
chr7	Lilra5	0.057094225	2	100.0000
chr3	Casq2	0.377452087	3	100.0000
chr3	Car14	0.397536108	2	100.0000
chr17	Rps10	0.476757358	2	100.0000
chr1	Rab7l1	0.499367019	4	100.0000
chr10	**Igf1**	0.546404576	2	100.0000
chr11	Srsf1	1.839627401	18	0.0000
chr16	Dlg1	1.843121706	19	5.2632
chr15	**Rictor**	1.850269418	31	9.6774
chr10	Ppa1	1.856936562	17	5.8824
chr10	Cs	1.871462757	13	0.0000
chr11	Psmd12	1.872473838	7	0.0000
chr1	Dst	1.893206535	15	0.0000
chr4	**Ctnnbip1**	1.901794587	20	5.0000
chr19	Smc3	1.905433481	23	4.3478
chr8	Naf1	1.915112216	13	0.0000
chr5	Limch1	1.947995552	15	0.0000
chr4	Hspg2	1.948056828	15	6.6667
chr17	Lclat1	1.980329242	15	0.0000
chr4	Sh3gl2	1.99214434	8	0.0000
chr3	Igsf3	2.016889197	22	0.0000
chr6	Aebp2	2.019027499	17	0.0000
chr7	Tshz3	2.030274352	48	4.1667
chr14	Hs6st3	2.040849538	37	0.0000
chr2	Mettl8	2.041718587	2	0.0000
chr16	Eif4g1	2.084266404	32	3.1250
chrX	Cdkl5	2.095452345	2	0.0000
chr13	Dip2c	2.110943596	11	0.0000
chr11	Ccdc88a	2.116618436	18	5.5556
chr1	Ncoa2	2.129957531	3	0.0000
chr5	Atxn2	2.171412892	35	5.7143
chr2	Tanc1	2.180687145	25	8.0000
chr4	E130308A19Rik	2.198178177	25	4.0000
chr17	Smchd1	2.209599662	3	0.0000
chr9	Pml	2.216518007	6	0.0000
chr5	Ttc28	2.221237478	13	0.0000
chr5	Lrrc8d	2.246820303	31	3.2258
chr18	**Apc**	2.24916978	17	0.0000
chr2	**Plcb1**	2.260103731	22	0.0000
chr9	Cspg4	2.272506555	4	0.0000
chr2	Rabgap1	2.290957971	12	8.3333
chr12	Srp54a	2.291532794	6	0.0000
chr17	Pgp	2.298921387	23	4.3478
chr16	Dopey2	2.345032587	17	5.8824
chr3	Fxr1	2.373885388	26	3.8462
chr12	Smoc1	2.416242757	18	5.5556
chr2	Cybrd1	2.475468964	14	0.0000
chr11	Plcd3	2.564426619	3	0.0000
chr12	Glrx5	2.611097466	26	7.6923
chr5	Ptpn12	2.64924452	4	0.0000
chr3	Sec62	2.700480763	5	0.0000
chr8	Gse1	2.747996289	36	8.3333
chr3	2810046L04Rik	2.790913895	11	0.0000
chr18	Mbp	2.791553334	2	0.0000
chr1	Cab39	2.843285092	21	0.0000
chr6	**Ccnd2**	2.853125022	8	0.0000
chr8	2310036O22Rik	3.340649265	14	7.1429
chr10	Ppp1r12a	3.800728332	28	3.5714
chr4	CK137956	3.848546303	4	0.0000

A final listing of genes with correlated changes to methylation and promoter methylation as described in Methods.

Note: Genes exhibiting both significant (p<0.05) and substantial (>1.5 fold change) change in expression in addition to more than methylation site observed within its promoter region with no more than 10% of observed sites differing in direction of methylation change. Genes with bold names are involved in the WNT or mTor pathway.

We conclude that acute sublethal hypoxia has a lasting impact on DNA methylation that persists days after normoxia is restored and that this modulation of methylation status, particularly in promoter regions and CpG islands, is correlated with the expression of neuronal genes involved in networks such as Wnt and mTOR that have been linked to roles regulating growth and development. The clinical significance of hypoxia to cell viability and functionality cannot be understated, and a deeper understanding of the mechanism of how hypoxia leads to changes in gene expression through epigenetic mechanisms will shed some light on the effect of hypoxia on gene expression and thus the phenotypic consequence of a stress like hypoxia.

## Supporting Information

Table S1 **Observed genome methylation status after filtering.** A listing of CCGG sites observed with MSCC assay after being filtered for reads and percentage change as described in Methods.
(XLSX)

Table S2 **Genes with significant expression changes.** A listing of genes with significant changes in expression based on RNASeq reads processed with the RUM aligner and DESeq differential expression analysis as described in Methods.
(XLSX)
